# Low-Rank and Eigenface Based Sparse Representation for Face Recognition

**DOI:** 10.1371/journal.pone.0110318

**Published:** 2014-10-21

**Authors:** Yi-Fu Hou, Zhan-Li Sun, Yan-Wen Chong, Chun-Hou Zheng

**Affiliations:** 1 College of Electrical Engineering and Automation, Anhui University, Hefei, China; 2 State Key Laboratory for Information Engineering in Surveying, Mapping and Remote Sensing, Wuhan University, Wuhan, China; University of Ulm, Germany

## Abstract

In this paper, based on low-rank representation and eigenface extraction, we present an improvement to the well known Sparse Representation based Classification (SRC). Firstly, the low-rank images of the face images of each individual in training subset are extracted by the Robust Principal Component Analysis (Robust PCA) to alleviate the influence of noises (e.g., illumination difference and occlusions). Secondly, Singular Value Decomposition (SVD) is applied to extract the eigenfaces from these low-rank and approximate images. Finally, we utilize these eigenfaces to construct a compact and discriminative dictionary for sparse representation. We evaluate our method on five popular databases. Experimental results demonstrate the effectiveness and robustness of our method.

## Introduction

Sparse representation algorithm has been successfully applied in image restoration [1] and compressed sensing [2] in the past several years. Recently, it has also led to promising results in image classification such as face recognition [3]–[5] and texture recognition [6]. For face recognition, given an over-complete dictionary, a testing face image can be linearly represented as a sparse coefficient vector. With the recent progress of *l*_0_-norm and *l*_1_-norm minimization techniques, classification based on sparse representation has became a hot topic owing to the fact that a high-dimensional image vector can be well mapped to a low-dimension manifold.

The sparse representation model can be succinctly denoted as the following optimization equation: 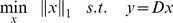
(1)Where 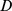 is an over-complete dictionary, 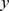 is a testing sample, e.g., a face image need to be identified, 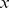 is the sparse coefficient representation vector over dictionary 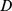.

In the early stage, some literatures [7]–[9] directly grouped the off-the-shelf bases together as the dictionary atoms. However, with the further research in sparse representation, learning an excellent dictionary has been proved to be an effective way to improve the capacity of signal reconstruction noticeably. To improve the performance of sparse representation, many algorithms have been proposed to optimize dictionary and acquired corking performance. E.g., Aharon et al. [10] generalized the k-means clustering process and proposed K-SVD algorithm, which iteratively updated the sparse representation coefficients based on the current dictionary and then optimized the dictionary atoms to better fit the data. Mairal et al. [11] presented an energy formulation with both sparse re-construction and class discriminative components. An on-line dictionary learning algorithm based on stochastic approximation was also developed to handle large datasets with millions of training samples [12]. In addition, Yang et al. [13] applied the fisher discrimination dictionary learning (FDDL) scheme to learn a discriminative dictionary for sparse representation.

These algorithms work well for the clean images or those images corrupted by slight noises. However, if both training images and testing images are corrupted by heavy noises, we should optimize the dictionary to better reconstruct the images including the noises. Although an identity matrix [4] can be introduced into dictionary to encode the corrupted pixels, it affects the sparsity of representation coefficients. On the other hand, supposing that the corrupted pixels are separated or suppressed so that images become clean as much as possible, and then use the clean data to learn a dictionary, the results of classification or reconstruction should be improved.

Recently, some algorithms have been proposed to separate the low rank and approximate data from original corrupted data. E.g., Candes et al. [14] alleviated the influence of noises and occlusions by Robust Principal Component Analysis (Robust PCA) algorithm. Liu et al. [15] generalized the Robust PCA and proposed Low-Rank Representation(LRR)to deal with the original data and utilized the low-rank structures to segment subspaces or classify face images. Long et al. [16] improved sparse representation for face recognition based on the discriminative low-rank dictionary learning method. Zhou et al. [17] applied Go Decomposition (GoDec) algorithm to extract the low-rank term quickly and obtained commendable performance in image and video processing. Thereby, we, in this paper, alleviate the influence of noises exiting in training samples according to the research about low rank representation.

For dictionary learning, another attractive aspect is the compactness of the constructed dictionary [18]–[20]. Zheng et al. [20] constructed a compact dictionary with meta-samples and regarded samples (genes expression data) as a linear mixture of independent basis snapshots (i.e., meta-samples). The results indicated that the compact dictionary achieved better recognition accuracy and decreased time consuming greatly. Actually, every face image could be represented by a large number of atoms in face space. We could extract these atoms (i.e., so-called eigenfaces) capturing the intrinsic structural information of each face space and use them to construct a compact dictionary for sparse representation.

Based on the aforementioned analysis, in this paper we incorporate low-rank and eigenfaces extraction into sparse representation for dictionary learning. Firstly, the low-rank images of training samples are extracted by Robust PCA to make the training images as clean as possible. Then, SVD is applied to extract eigenfaces from these low-rank and approximate images. Finally, these eigenfaces are combined to construct a compact dictionary for sparse representation based classification. The proposed algorithm can not only separate the influence of sparse noises but also enhance the compactness of dictionary. Compared with other dictionary learning algorithms, the merits of the proposed approach are listed as follows: First, we apply low rank transformation to compress the noises existing in training images so that the extracted features are more discriminative for classification. Second, we extract eigenfaces from the low-rank face images by mathematical method to make the dictionary more compact. Third, comparing with the classical dictionary learning algorithms, our method is robust to some large but sparse errors such as block occlusions.

The remainder of the paper is organized as follows: Some related works about low-rank representation are introduced in Section 2. Section 3 presents our algorithm in detail. Section 4 shows the experimental results. And some theoretic analysis are also listed in this Section. Section 5 concludes the paper and outlines the future work.

## Low-Rank Representation Algorithm

### Robust PCA for Low-Rank Matrices Recovery

How to exploit low-rank structure from high-dimensional data is taking on increasing attention in image, audio and video processing. Meanwhile, it is also a challenging task to exactly recover the low-rank structure from the high-dimensional and corrupted data. The application of classical Principal Component Analysis (PCA) [21] suffers from a prodigious limitation owing to its brittleness with respect to serious corrupted data. Although some approaches such as multivariate trimming [22], alternating minimization [23] and random sampling techniques [24] can improve the robustness of PCA, none of them obtains a polynomial-time algorithm with strong performance guarantee. Motivated by the recent research on robust solutions of over-determined linear systems and low-rank solutions of under-determined linear systems, Candes et al. [14] proposed the Robust PCA algorithm which decomposed a corrupted matrix into a low-rank matrix and a sparse errors matrix.

Assuming that a cleaning data matrix 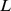 is corrupted by noises (i.e., error term 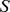) and becomes the corrupted data matrix 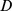, i.e., 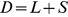
(2)where 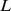 and 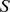 are unknown but 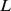 is a low rank matrix and 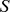 is sparse. The process of recovering 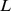 from 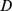 can be reformulated as the following optimization problem:
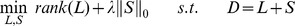
(3)where 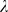 indicates the percentage of sparse errors. Unfortunately, it is a highly non-convex optimization problem so that no efficient solution can be obtained. We relax formula (3) (i.e., replacing the 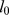-norm and the rank term with the 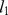-norm and the nuclear norm, respectively) and yield the following convex function:
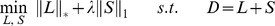
(4)the minimized solutions (denoted as 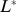 and 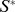) are named as the low-rank part and sparse errors part of the corrupted matrix 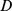.

### Augmented Lagrange Multiplier (ALM) Method for Robust PCA

We adopt in this paper the Augmented Lagrange Multiplier (ALM) [25]–[26] to solve problem (4). Generally, the ALM algorithm is used to solve the following optimization function: 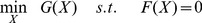
(5)where 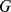 is a convex function, 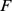 is a linear function. Equation (5) is solved by defining augmented Lagrange function:
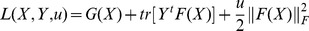
(6)where 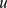 is a positive scalar, 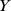
is a Lagrange multiplier vector. Correspondingly, the augmented Lagrange function of Equation (4) is:
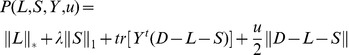
(7)here, 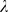 is a parameter and generally computed through the following formula:
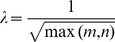
(8)where *m* and *n* are the number of columns and rows of matrix 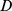, respectively. In the experiment section, we will show how to select the optimal 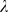 for face matrix 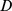. The parameter 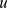 in Equation (7) can be adjusted by the following strategy:
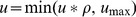
(9)where 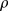 satisfies the condition 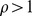 (e.g., 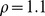) to control the velocity of the increase of 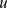. 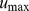 is the upper bound of 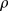 (e.g., 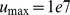).

The optimization for Equation (7) can be divided into two sub-problems. The first sub-problem is to compute 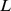 for a fixed 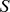. The second sub-problem is to solve 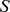 for the fixed 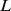 computed from the first sub-problem. We update 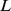 and 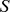 as follows:
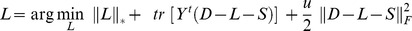





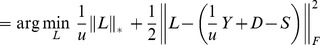
(10)




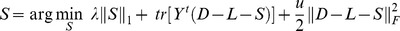





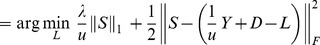
(11)


The ALM algorithm for Robust PCA is summarized as follows:

Algorithm: ALM algorithm for Robust PCA [27]

Input: Corrupted matrix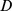,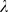,
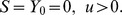


Step1: while not converged do

Compute 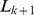 according to formula (10);

Compute 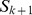 according to formula (11);

Compute 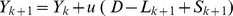;

End while

Output: 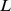,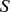.

## The ESRC_LR Based Face Recognition Algorithm

### Eigenfaces of Face Images

**Figure 1:**
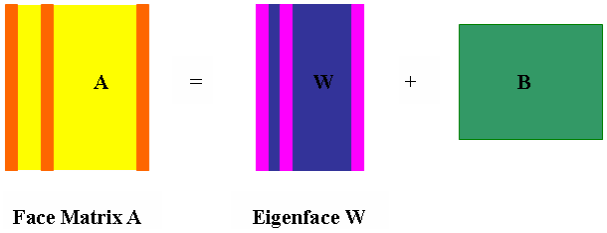
The relation between face matrix and eigenface matrix. Each column of matrix 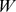 represents a eigenface, and each sample (column) in face matrix 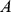 can be represented by eigenfaces in eigenface matrix 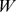 with eigenface expression pattern (column in matrix 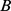) of the corresponding sample.

Generally speaking, eigenfaces of face images are defined as several special faces which capturing intrinsic structural information of face space. They are similar to a group of bases of linear space in matrix theory. For sparse representation, the number of training samples may be considerable. This probably results in redundancy of the constructed dictionary and more time expending during practical applications. Actually, by applying mathematical methods to extract eigenfaces and combining them to construct a compact dictionary for sparse representation, we still can obtain a sparse solution by solving 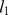-norm minimization problem [28] for each testing sample. Mathematically, the face images dataset matrix 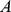 can be decomposed as the product of two matrices:
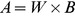
(12)where matrix 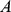 is of size 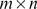 with 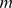 dimensions and 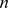 samples, matrix 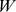 is of size 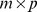 and each of the 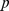 columns is defined as an eigenface, matrix 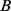 is of size 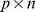 and each of the 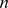 columns represents eigenface expression pattern of corresponding sample. Figure 1 shows the relation between face matrix 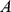 and eigenface matrix 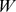.

The methods about how to extract eigenfaces (eigengenes or metasamples) have been published in many literatures [29]–[31]. E.g., Alter et al. [29] extracted eigengenes by SVD to transform the “genes × samples” space to the diagonalized “eigengenes × eigenarrays” space. Zheng et al. [30] applied Independent Component Analysis (ICA) to model genes expression data regarding samples as a linear mixture of independent basis snapshots (i.e., metasamples). Brunet et al. [31] similarly utilized a small number of metasamples extracted by Non-negative Matrix Factorization (NMF) to represent testing samples. Consequently, we can extract eigenfaces from face data by means of several methods such as SVD, ICA and NMF.

### Eigenface-based Sparse Representation Classification

Owing to the characteristic of eigenfaces, in this paper we extract eigenfaces from face images of each class, respectively, and then combine them instead of the original training data to design a compact dictionary for sparse representation. In practice, each sub-dataset (i.e., including only one class) 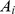 is factorized into two matrices:
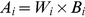
(13)where 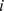 represents the 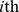 class samples. Matrix 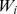 is of size 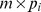 and each of the 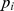 columns represents an eigenface.

After extracting the eigenface matrix 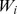 from each class, we combine them to build a collaborative dictionary 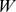 for sparse representation, i.e., 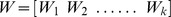, where 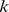 represents the number of classes. Now, given a testing sample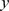, we compute its sparse representational solution over the collaborative dictionary, i.e., 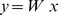. Ideally, 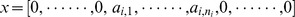 is a sparse vector whose entries are zeros except for these associating with the 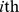 class. We identify the class label to which a testing sample belongs by analyzing its nonzero entries of coefficient representation vector [4], [28].

Obviously, for a 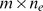 matrix 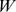
(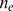 represents the total number of eigenfaces), if 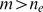, 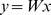 is over-determined and its correct solution is unique. But in the face recognition system, 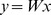 typically represents under-determined system, and its solution is not unique. To achieve a sparse solution, we adopt 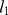-norm minimization:
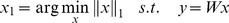
(14)The standard linear programming methods is applied to solve (14) in polynomial time. Moreover, to certain degree of noises, we adopt the following objective function to solve 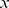, i.e.,
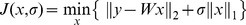
(15)where the positive parameter 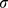 is a scalar regulator.

In practice, model errors and certain degree of noises inevitably cause some nonzero entries associating with different classes, which may bring about misclassification. To design a more robust classifier, we compute reconstruct residual as the basis of judgment [4], i.e., 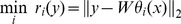
(16)where 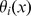 is a vector whose nonzero entries are these from class 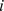 in 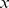.

The eigenface-based sparse representation classification algorithm is described as follows:

Input: Matrix of training samples 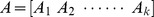
(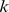
classes); a testing sample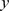.

Step1: Extract the eigenfaces matrix 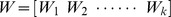 by SVD;

Step2: Solve the optimization problem defined in (15);

Step3: Compute the residuals through formula (16);

Output: 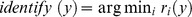.

### Overview of Our Method

**Figure 2:**
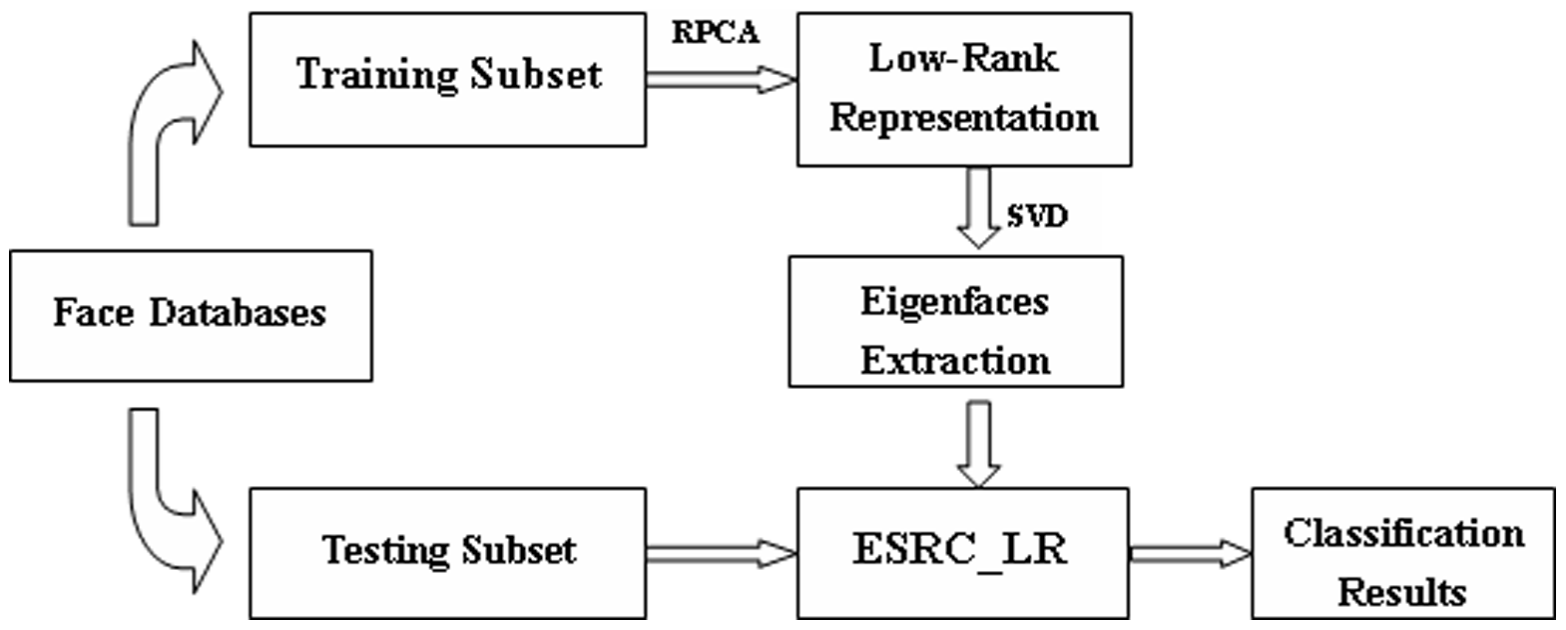
Overview of our method.

Figure 2 shows the overview of our method. For a face database, we firstly divide it into two parts, i.e., the training subset and the testing subset. Then, we extract low rank images for each class images in training subset by the Robust PCA, which will make the training images as clean as possible. Next, SVD is applied to extract the eigenfaces from the clean images. These eigenfaces are used as a compact dictionary for sparse representation based classification. We named our strategy as Low-Rank and Eigenface based Sparse Representation Classification (i.e., ESRC_LR). Finally, we apply the proposed ESRC_LR to classify all of the testing samples.

Since the face images are captured in real world and more or less corrupted by some negative factors such as varying illumination, shadowing, uniform noises and even random block occlusions, we had better adopt a preprocessing operation to acquire clean data as much as possible. Aiming at alleviating or eliminating the influence of negative factors, we extract the low-rank images by Robust PCA to get relatively more clean face images. In addition, we apply SVD to extract eigenfaces from the corresponding low-rank images. These eigenfaces could all-rightly describe intrinsic structural information of corresponding face spaces. By combining all of eigenfaces to construct a compact dictionary, we can improve the performance of standard sparse representation based classification algorithm. In practice, to alleviate the influence of noises and model error, we classify a testing sample based on how well it can be reconstructed by the representational coefficients of corresponding class.

The proposed ESRC_LR algorithm is summarized as follows:

Input: matrix of training samples 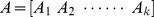 (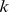
classes); a testing sample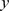.

Step1: while 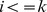 do
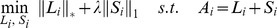
end while
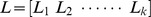
Step2: while 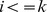 do 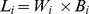
end while 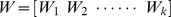
Step3: Solve the optimization problem 
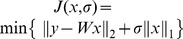
;

Step4: Compute the residuals 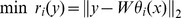;

Output: 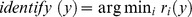;

## Experimental Results

Firstly, in this section, we conduct experiments to illustrate how to make an optimal choice for parameter 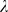 in Equation (4) and the influence of different number of eigenfaces to recognition rate on the extended Yale B Database [32]. Secondly, we apply the proposed ESRC_LR algorithm to face recognition on four publicly available face datasets, i.e., the extended Yale B Database, the AR Database [33], the ORL Database [34] and CMU-PIE Face Database [35]. Thirdly, we evaluate the robustness of ESRC_LR algorithm to different percentage of uniform noises and block occlusions. The summary of utilized databases are listed in Table 1:

**Table 1:** The information of utilized databases.

Datasets	number of classes	samples per classes	number of features	train samples/test samples(per class)
**the Extended Yale B dataset**	38	64	504 (downsample ratio:1/8)	40/24
**the AR dataset (part)**	100	26	1600(40*40)	7/7(without occlusion, each session)
**the ORL dataset**	40	10	750(30*25)	8/2
**the CMU-PIE dataset**	68	49	4096(68*68)	25/24(almost equal)
**The UMIST dataset**	15	almost 28	600(30*20)	almost equal

To show the effective of our method, we also used other methods to classify the datasets for comparison. They are Fisher Discrimination Dictionary Learning algorithm (FDDL) [13], Sparse Representation based Classification (SRC) [4], Support Vector Machine (SVM) and Nearest Neighbor (NN). In addition, during all of the following experiments, we set the number of iterations to 25 for learning dictionary in FDDL. Parameter 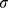 in formula (15) is fixed to 0.15 for SRC according to [20]. SVM with different kernel function (i.e., linear kernel and RBF kernel) is applied and Nearest Neighbor with K = 1.

### The Choice of Parameters

#### Tuning Parameter 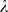 for Face Matrix 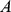


In Equation (4), 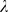 presents the percentage of sparse errors. The low-rank representation results are extremely sensitive to parameter 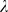. Although we could compute it using Equation (8), it is not optimal. In practice, we initialize 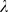 by Equation (8) and then increase or decrease 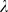 step by step at interval of 0.005 to choose the best one. We used the experiment on extended Yale B Database to adjust parameter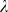. The former 40 images were selected as training images and the remaining as testing images, and all of the images were down-sampled with ratio 1/8.

**Figure 3:**
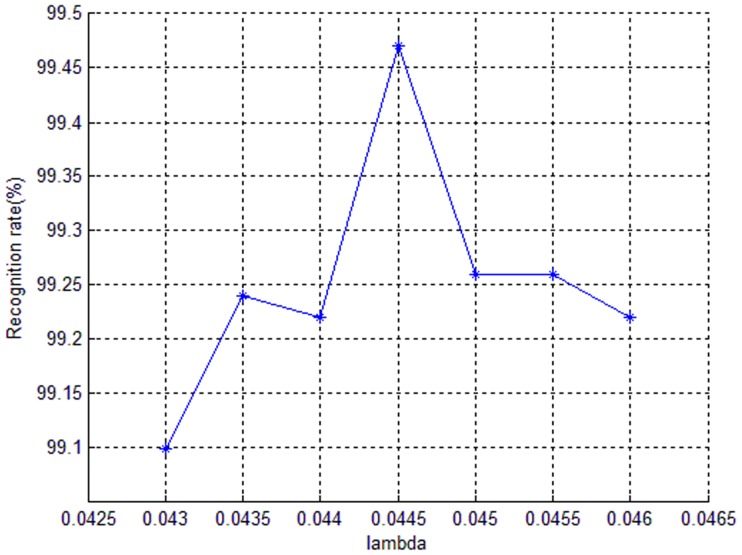
The relation between recognition rates and the corresponding parameter 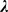.

Figure 3 shows the relation between recognition rates and the corresponding parameter 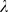. From this figure we can confirm that 0.0445 is the best choice for 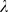, since the recognition rate is the hightest one with this value. Through this way we can also obtain the best 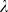 for other different databases.

#### Influence of Different Number of Eigenfaces

**Figure 4:**
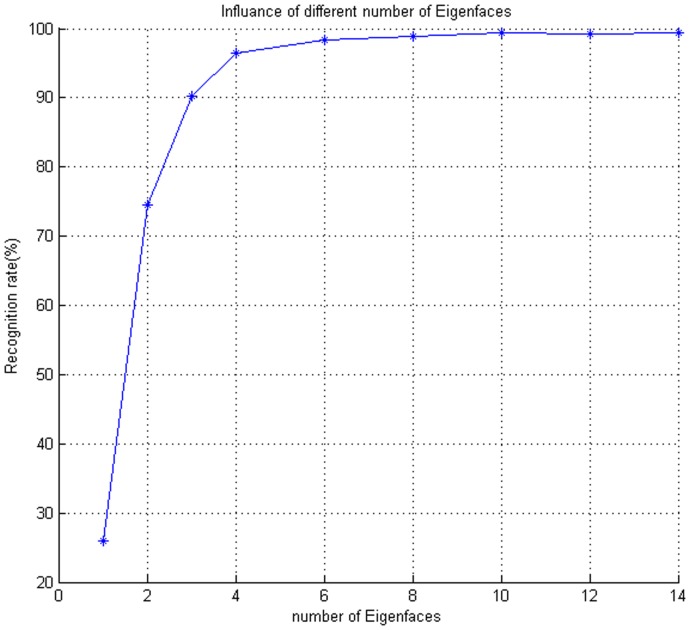
Accuracy rate of different number of eigenfaces. The number of eigenfaces 1, 2, 3, 4, 6, 8, 10, 12, 14 correspond to 25.95%, 74.59%, 90.30%, 96.46%, 98.31%, 98.89%, 99.47%, 99.30%, 99.47%.

As mentioned above, eigenfaces can capture intrinsic structural information of face space, so different number of eigenfaces performs different performance. Need not go into detail that overmuch eigenfaces result in much time expending in applications, and too few may be insufficient to express integrity of face space, classification accuracy is not guaranteed as a consequent. Experimental results about the performance of different number of eigenfaces on extended Yale B Database are listed in Figure 4. We set 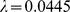 to extract the low-rank images of training samples by Robust PCA algorithm.

Figure 4 shows that the recognition rate goes up promptly with the increasing of the number of eigenfaces, and it is low when the number of eigenfaces is less than three. The reason should be that minority eigenfaces cannot integrally capture inherent structural information of face space. Therefore, some testing samples can not be well reconstructed by these eigenfaces. After greater than 4, with the further increase of the number of eigenfaces, recognition rate rises slowly and reaches a relatively steady point, which illustrates the selected eigenfaces are enough to span face space. Certainly, to different face databases, the suited number of eigenfaces still needs to conduct experiments to determine.

### Face Recognition without Occlusions

In this subsection, we apply the proposed ESRC_LR algorithm to classify face images on four popular databases (i.e., the Extended Yale B Database [32], the AR Database [33], the ORL Database [34] and CMU-PIE Face Database [35]), and compare it with FDDL[13], SRC[4], SVM with linear kernel function and RBF kernel function and NN with K = 1.

#### Evaluation on the Extended Yale B Database

The Extended Yale B Database consists of 2414 frontal-face images of 38 individuals. The images were captured under various laboratory-controlled lighting conditions. We selected the former 40 images as training images and the reminder as testing images and all of the images were down-sampled with ratio 1/8. In the experiment we chose the popular methods such as SRC, SVM, NN and the recently published FDDL for comparison. The recognition rates are listed in Table 2.

**Table 2:** The recognition rates on Extended Yale B Database.

Algorithms	ESRC_LR	FDDL	SRC	SVM (RBF)	SVM (linear)	NN
**Recognition rate(%)**	99.47	93.31	89.95	88.25	78.25	77.83

**Figure 5:**
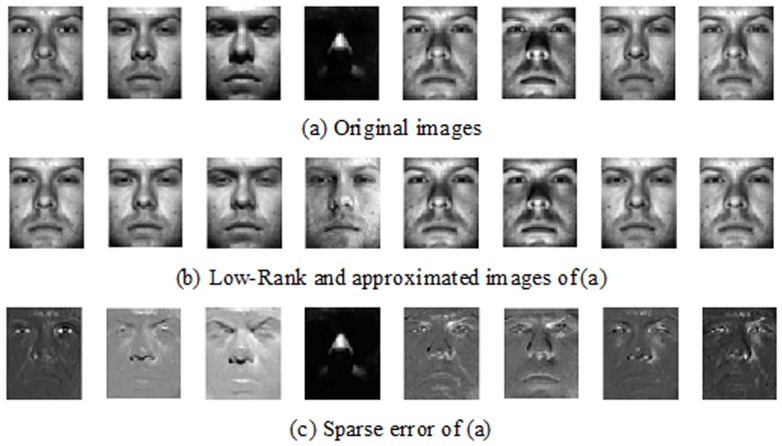
An example of Robust PCA algorithm on the Extended Yale B Face Database. First row is the original images with vary illumination and expression changes. Second row shows the low rank and approximate images of (a). Third row is the sparse error images of (a) which is the difference of (a) and (b).

**Figure 6:**
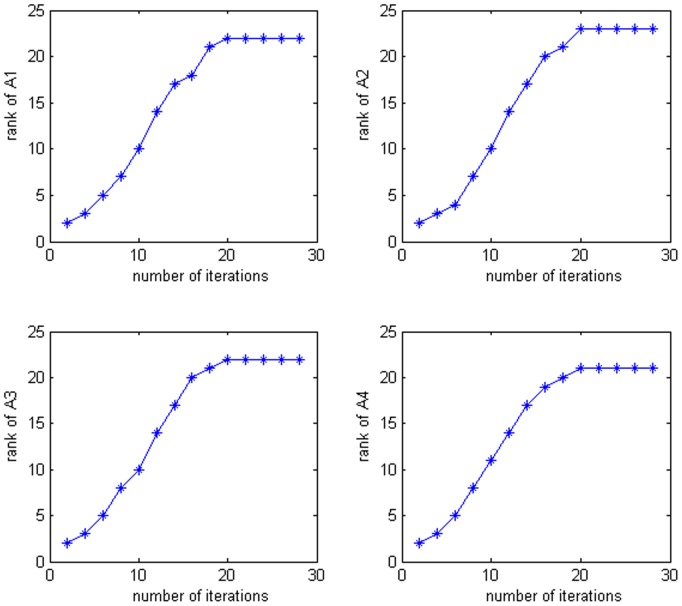
The relation between ranks of face matrices and number of iterations. Top-left represents the relation between the rank of first class face matrix and the number of iterations on Yale B Face Database with 40 training samples. Top-right, bottom-left and bottom-right represent that of second, third and forth class face matrices, respectively.

Figure 5 shows an example of the extracted low-rank and approximate images on Extended Yale B Database. It is easy to find that the low rank images are similar to original images but illumination difference and expression changing have been alleviated. Figure 6 illustrates the relation between the ranks of face images of four classes and the number of iterations. We can see from figure 6 that the low rank algorithm is convergent. The rank of each class is stabilized at about 22 finally, which is much smaller than the rank of original corrupted face images (i.e., about 40). The reduction of rank leads to the alleviation of illumination difference and expression changing. In the experiment we selected 14 eigenfaces from each class according to subsection 4.1, and set 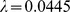 to extract the low rank images. Comparing with other classical methods shown in Table 2, the proposed ESRC_LR algorithm exhibits better performance than FDDL, SRC, SVM(RBF), SVM(linear) and NN(K = 1).

#### Evaluation on the AR Database

The AR face database consists of over 4000 frontal images with 126 individuals. For each individual, 26 pictures were composed of two sessions. In each session, 7 pictures were about varied expression, 3 pictures were covered by mask and the rest 3 pictures wore sunglasses. In our experiment we chose 50 males and 50 females and all of images were cropped to 40×40 pixels and converted to grayscale. For each individual, the 7 images with illumination and expression changes from session 1 were used for training, and the other 7 images with the same changes from session 2 were chosen for testing. We fixed 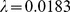 for extracting the low rank representation of face matrix, and selected 4 eigenfaces for each class to construct a compact dictionary. The comparison between several competing methods is shown in Table 3. It can be seen that the proposed algorithm (recognition rate 97.66%) improves about 6.37% over the second best performance (FDDL: 91.29%), and 8.53% over the third best performance (SRC: 89.14%). The experimental results show that the low-rank processing and eigenfaces extraction can do improve the performance of dictionary, thereby, coding the query image over the learnt dictionary is more reasonable. From this experiment, we can also draw a conclusion that our method is quite effective to deal with illumination difference and expression changing.

**Table 3:** The recognition rates on AR Face Database.

Algorithms	ESRC_LR	FDDL	SRC	SVM (RBF)	SVM (linear)	NN
**Recognition rate (%)**	97.66	91.29	89.14	73.14	66.00	75.94

Recent years, the kernel optimization based algorithms are popular and competitive. It is necessary to compare our algorithm with some kernel optimization based algorithms mentioned in [36] on the AR database. For simplification, we also resized the images into 29×21 pixels. The images in session 1 were used for training and those in session 2 for testing. One could refer to [36] for details. The results are shown in Table 4 (some results originate from [36] directly). As we all known, kernel discriminant analysis (KDA) maps the original data with unequal covariances into a homoscedastic space. However, to a multi-model structure in each class, KDA may be limited to smooth solutions which would result in large classification error. In our method, we try to seek a better representation over a learnt dictionary for each testing sample (i.e., learning a dictionary with low-rank processing and eigenface extraction). By comparison, our method achieves slightly better results than KDA. However, to multi-model class representations, kernel based subclass DA(KSDA) can find some smoother functions than KDA so as to carry much smaller classification error (in some case, surpass our algorithm a lot).

**Table 4:** The comparison of kernel optimization based algorithms with our algorithm.

	KSDAH	KDAH	KNDAH
**Nearest Mean**	88.1	87.5	71.3
**Nearest Neigbor**	96.7	88.3	69.2
**Linear SVM**	88.1	87.5	79.4
**Method in** [37]	96.6	90.6	70.9
**ESRC_LR**	89.27		

KSDA, KDA and KNDA present kernel discriminant analysis, kernel subclass discriminant analysis and kernel non-parametric discriminant analysis, respectively. Subscript ‘H’ presents homoscedastic-based optimization.

#### Evaluation on ORL Face Database

The ORL database contains 400 images in total, each of 40 individuals has ten different images shot under different times, varying the lighting, facial expressions and facial details. The background of the images is uniform while the subjects are in frontal, upright positive. For each individual, we randomly selected 8 images as training samples and the rest as testing samples and repeated the experiment 10 times. All of images were normalized to 30×25 pixels. We fixed 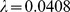 for extracting the low rank representation of face matrix, and selected 6 eigenfaces for each class to construct a compact dictionary. The average recognition rates are shown in Table 5. According to this Table we can easily see that the recognition rate of ERSC_LR reaches 96.88% which outperforms FDDL (95.31%) and SRC (95.50%) and far better than SVM(RBF) (85.00%), SVM(linear) (81.87%) and NN(K = 1) (72.75%). The ESRC_LR algorithm has at least 1.3% improvement over the second best performance, which also illustrates that our method is more powerful than other methods.

**Table 5:** The recognition rates on ORL Face Database.

Algorithms	ESRC_LR	FDDL	SRC	SVM (RBF)	SVM (linear)	NN
**Recognition rate (%)**	96.88	95.31	95.5	85.00	81.87	72.25

#### Evaluation on CMU-PIE Face Database

**Figure 7:**
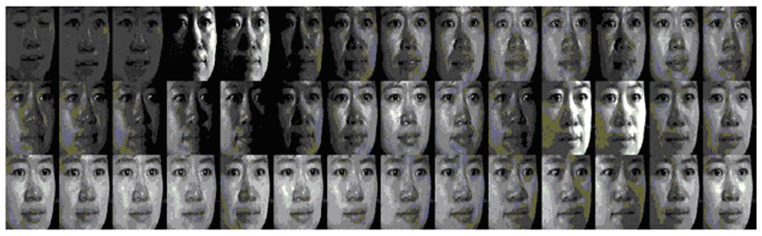
Some examples from the CMU-PIE Face Database with variation in illumination, expression.

The CMU-PIE database contains about 41,368 images of 68 distinct individuals. The images of each subject are captured under different poses, variable illumination conditions, and with different expressions. In this paper, one of near frontal pose subsets, namely C05, is chosen for experiments. There are 3332 images in total with size of 64×64 pixels which already detect the face and eyes regions from the original images. Figure 7 shows an example of images in the subset. For each person, we randomly selected about half number of images as training samples and the rest as testing samples and repeated each experiment 10 times. We fixed 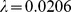 for extracting the low rank representation of face matrix, and selected 20 eigenfaces for each class to construct a compact dictionary. The average recognition results of different approaches are shown in Table 6. From which, we can see that the recognition rate of ERSC_LR algorithm (97.97%) is better than SVM (linear) (94.37%) and NN (90.28%) but similar to FDDL (97.01%), SVM (RBF) (97.72%) on CMU-PIE Face Database. Actually, SVM with RBF kernel function nonlinearly maps the original face data into a high-dimensional space to make linearly impartible problem divisible, so the performance of RBF kernel function is superior to linear kernel function according to our experimental results. Notice that the mapped data in high-dimensional space may still be linearly impartible due to the curse of dimensionality. However, in our algorithm, each testing sample is represented by eigenfaces extracted from low-rank images as a coefficient vector so that it is easier for classification than that extracted from the corrupted data. Although FDDL uses of the fisher discrimination criterion to learn a structured dictionary, the large-scale sparse errors in training samples limit its performance. In fact, our method and FDDL are both improved methods based on standard SRC. Table 6 shows that our method obtains slightly better recognition accuracy than SVM with RBF kernel function and FDDL.

**Table 6:** The recognition rates on CMU-PIE Face Database.

Algorithms	ESRC_LR	FDDL	SRC	SVM (RBF)	SVM (linear)	NN
**Recognition rate (%)**	97.97	97.01	97.89	97.72	94.37	90.28

**Figure 8:**
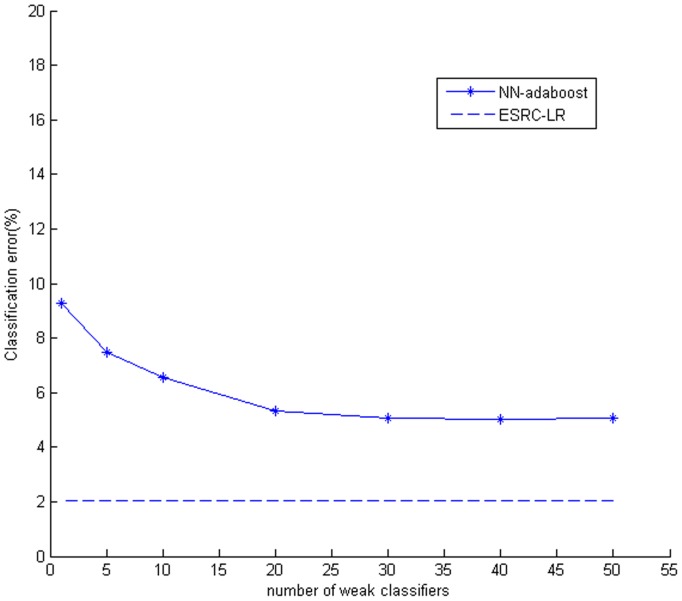
Classification errors under different number of weak classifier.

Recently, the cascade classifier, another kind of face recognition system, is popular. Shan et al. [38] proposed the AdaBoost Gabor Fisher Classifier for robust face recognition, in which a chain Adaboost learning method based on Booststrap re-sampling was proposed and applied to face recognition. To compare ESRC_LR algorithm with the cascade classifier based face recognition system, we made another experiment on the CMU_PIE face dataset. The popular nearest neighbor based adaptive boosting (NN_adaboost) classifier was applied and we also set the same experimental condition as above. Different number of weak classifiers was cascaded and the corresponding classification results were shown in Figure 8.

From Figure 8 we can see that classification error decreases gradually with the increase of number of weak classifiers. In the experiment, the classification error of single NN classifier is 9.72% according to the above experiment. After cascading 50 NN classifiers, the classification error drops to about 5%, Owing to the augment of weight for misclassified samples to make them classify easily in each training process of weak classifier, the final strong classifier (cascade of weak classifiers) achieves much better performance. However, experimental result also shows our ESRC_LR algorithm (classification error: 2.03%) outperforms the NN_adaboost algorithm in spite of increasing the number of weak classifiers (classification error is no longer change apparently for more than 30 weak classifiers).

### Face Recognition with Occlusions

One of the most fascinating features of sparse representation based algorithms is the robustness to occlusions and sparse noises. Since they universally exist in both training and testing images, in this subsection we investigate the robustness of our algorithm to random distributed noises and random block occlusions. Firstly, we evaluate the robustness to random pixel corruptions on the AR Database. Secondly, the UMIST Face Database [39] is adopted to test the robustness to random block occlusions. We still choose previous methods (FDDL, SRC, SVM (linear and RBF), NN(K = 1)) as the baselines.

#### Robustness to Random Noises

**Figure 9:**
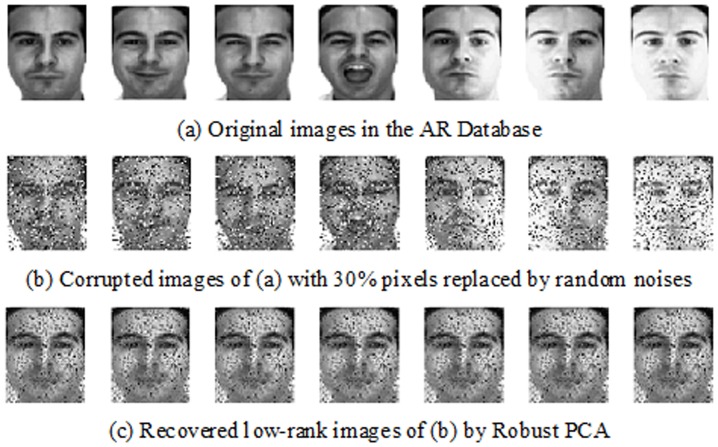
An example from the AR Database with 30% pixels corruptions. The top row is the original images. The middle row shows corrupted images of (a) with 30% pixels replaced by random noises. The noise values are random selected from [0, 255] and the locations are unknown. The below row is the recovered low-rank images of (b) by Robust PCA algorithm.

**Figure 10:**
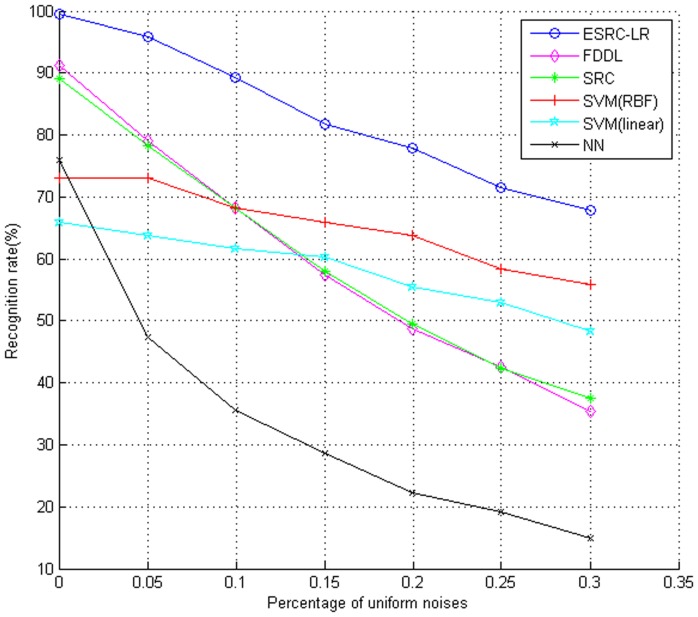
Recognition accuracy on the AR Database with different percentage of pixels corruptions.

Firstly, we investigate the robustness of our algorithm to random noises on the AR Database. For each individual, the seven clean images from Session 1 were selected for training, and the seven images from Session 2 were chosen for testing. All of the images were resized into 40×40 pixels and a certain percentage of pixels were replaced by uniformly distributed random values within [0, 255]. The corrupted pixels were randomly chosen in both training images and testing images and the locations were blind to algorithms. Figure 9 shows an example of face images corrupted by random pixel noises and the corresponding low-rank images extracted by Robust PCA algorithm. The recognition rates under different level of noises are given in figure 10. Obviously, all classification performance decreases with the increasing random noises. Moreover, the advantage of ESRC_LR algorithm over the other popular methods is clear. With higher random noises, the recognition rate of ESRC_LR algorithm decreases slowly, and outperforms FDDL and SRC algorithm by 15% improvement on average.

As we can see from the above experiment that the combined performance of low-rank estimation and eignefaces extraction outperforms the other popular algorithms evidently such as FDDL, SRC, SVM(linear and RBF) and NN(K = 1). To illustrate the separate effect of the two sub-processes, i.e., low-rank matrix estimation only and eigenfaces extraction only, we also conduct an experiment to research independent effect of the two sub-processes on the AR database with increasing corrupted pixels. The scenarios are: (i) low rank estimation followed by eigenfaces extraction, (ii) low-rank matrix estimation only, and (iii) eigenfaces extraction only. Figure 10 presents the experimental results of three different scenarios.

**Figure 11:**
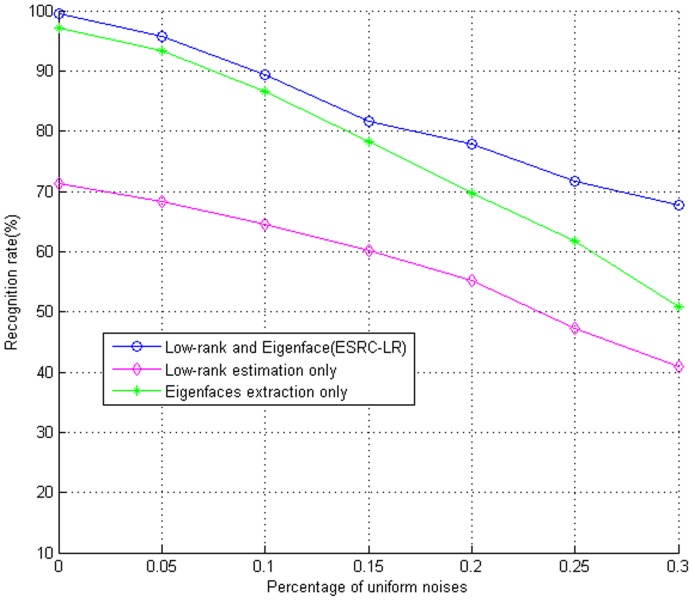
The separate effect of these two sub-processes of ESRC_LR algorithm on the AR Database with increasing corrupted pixels.

The comparison results between ESRC_LR and its two sub-processes (i.e., low-rank matrix estimation only and using eigenfaces only) are shown in figure 11. From this figure we can see that the proposed ESRC_LR algorithm outperforms its two sub-processes. With lower random noises, the ESRC_LR algorithm performs better than independent eigenfaces by about 2% improvement but far better than low-rank estimation. With the increase of random noises, the ESRC_LR algorithm is superior to the two sub-processes more obviously. The experiment also illustrates that the two sub-processes have a positive effect on the combined ESRC_LR algorithm.

#### Robustness to Random Block Occlusions

**Figure 12:**
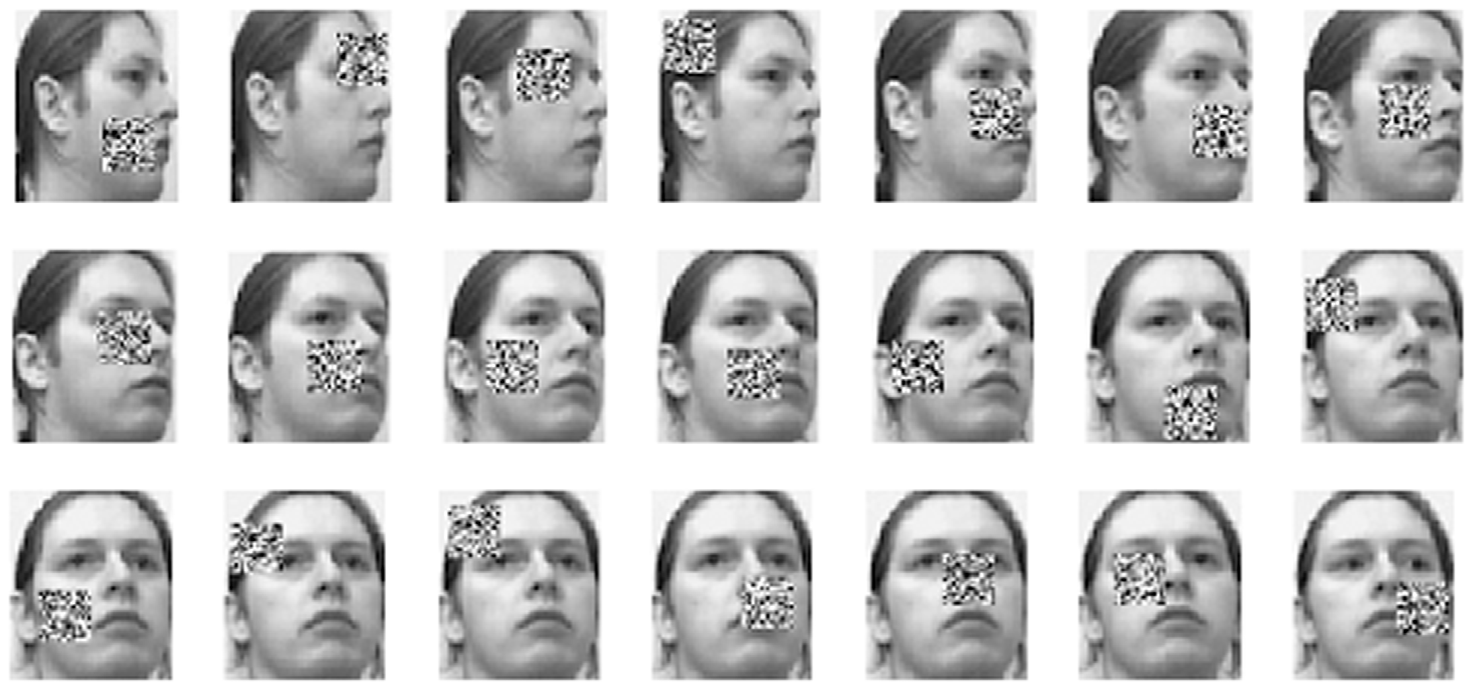
An example of images from UMIST Face Database with random block occlusions (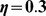).

**Figure 13:**
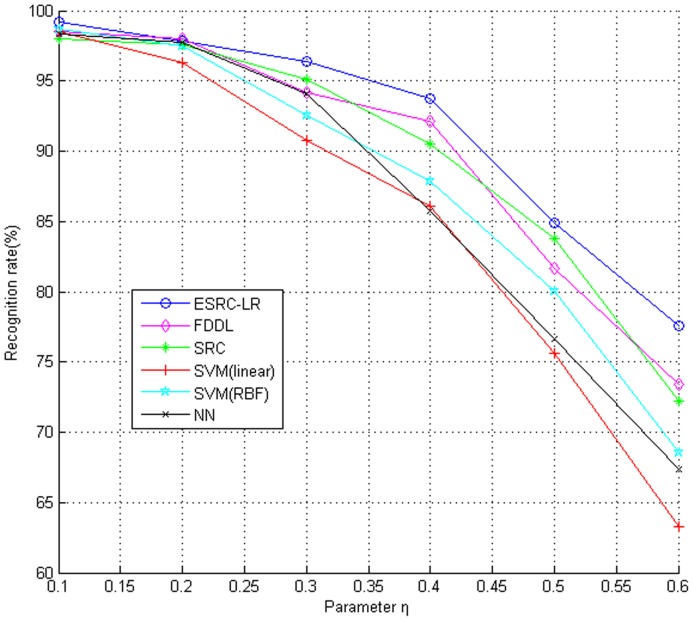
Recognition rates on UMIST Face Database with different level of block occlusions.

In this subsection we test the robustness of our method to random block occlusions on UMIST face database. The UMIST Face Database consists of 564 images of 20 individuals and each individual is shown in a range of poses from profile to frontal views. In the experiment, 15 individuals' images were used and all of images were resized into 30×20 pixels. We replaced a randomly located block of each image with an unrelated random image. The values in each block were randomly chosen from [0, 255] and the position of block in each image was randomly selected. A parameter 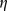 was set to control the size of block which met the following relations:
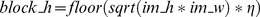
(17)




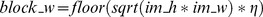
(18)where 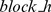 and 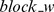 were the height and width of block, 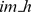 and 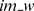 were the height and width of images, respectively. Figure 12 shows an example of training images and testing images with random block occlusions (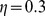) from UMIST Face Database. We adopted 4-fold cross validation to evaluate our algorithm. The recognition rates under different level of occlusions are given in figure 13. We can see the proposed ESRC_LR algorithm performs better than the other popular algorithms such as FDDL, SRC, SVM(RBF), SVM(linear) and NN(K = 1). For small block corruptions, the advantage of ESRC_LR algorithm is not obvious comparing with the other methods. However, as the block occlusion becoming larger, our ESRC_LR algorithm decreases slowly and outperforms other algorithms more and more apparent. Which shows the strong robustness of our method to random block occlusion.

## Conclusions

In this paper, we present an improvement to the well known Sparse Representation based Classification for face recognition. We firstly extract low rank images for each class in training subset to alleviate the influence of noises such as illumination difference and occlusions. Then, SVD is applied to extract eigenfaces from the clean face images. These eigenfaces are organized to construct a compact but discriminative dictionary for sparse representation based classification. We evaluate the proposed ESRC_LR algorithm under different conditions, i.e., clean images, uniform distributed noises and random block corruptions. Experimental results verify that our ESRC_LR algorithm is advantage and robust. However, how to alleviate the influence of large but sparse noises is still worth deep studying. Moreover, A new angle of view about how to extract effective feature on low-rank images to construct a better dictionary is worthy of consideration.
