# Correction: Low-Rank and Eigenface Based Sparse Representation for Face Recognition

**DOI:** 10.1371/journal.pone.0117902

**Published:** 2015-03-05

**Authors:** The PLOS ONE Staff

In the Funding section, the grant number 61370109 was omitted from the National Science Foundation of China. Please view the complete, correct Funding statement here.

This work was supported by the National Science Foundation of China under Grant Nos. 61272339 & 61370109, the Natural Science Foundation of Hubei Province under Grant No. 2012FFB04204, and the Key Project of Anhui Educational Committee, under Grant No. KJ2012A005. The funders had no role in study design, data collection and analysis, decision to publish, or preparation of the manuscript.
